# ESBL-Producing *Enterobacteriaceae*: Occurrence, Risk Factors for Fecal Carriage and Strain Traits in the Swiss Slaughter Cattle Population Younger than 2 Years Sampled at Abattoir Level

**DOI:** 10.1371/journal.pone.0071725

**Published:** 2013-08-20

**Authors:** Martin Reist, Nadine Geser, Herbert Hächler, Sara Schärrer, Roger Stephan

**Affiliations:** 1 Veterinary Public Health Institute, Vetsuisse Faculty University of Bern, Bern, Switzerland; 2 Institute for Food Safety and Hygiene, Vetsuisse Faculty University of Zurich, Zurich, Switzerland; Federal University of Pelotas, Brazil

## Abstract

During the past decade extended-spectrum beta-lactamase (ESBL) producing *Enterobacteriaceae* have become a matter of great concern in human and veterinary medicine. In this cross-sectional study fecal swabs of a geographically representative number of Swiss cattle at slaughterhouse level were sampled i) to determine the occurrence of ESBL producing *Enterobacteriaceae* in the Swiss slaughter cattle population younger than 2 years, and ii) to assess risk factors for shedding ESBL producing *Enterobacteriaceae*. In total, 48 (8.4%; 95% C.I. 6.3–11.1%) independent ESBL producing *Enterobacteriaceae* were detected among the 571 tested animals. Species identification revealed 46 *E. coli* strains, one *Enterobacter cloacae* and one *Citrobacter youngae.* In view of beta-lactam antibiotics, all 48 isolates were resistant to ampicillin, cephalothin and cefpodoxime. Forty-five (93.8%) isolates were resistant cefuroxime; one (2.1%) isolate to cefoxitin, 28 (58.3%) isolates to cefotaxime, 2 (4.2%) isolates to ceftazidime, and 2 (4.2%) isolates to cefepime. Risk factors for shedding ESBL producing *Enterobacteriaceae* were (i) age (OR 0.19 and 0.12 in age category 181 d to 1y and 1y to 2 y compared to ≤180 d), (ii) primary production type, meaning dairy compared to beef on farm of origin (OR 5.95), and (iii) more than 1 compared to less than 1 animal movement per d per 100 animals on farm of origin (OR 2.37).

## Introduction

Antimicrobial resistance in bacteria has emerged as a problem in both human and veterinary medicine. One of the currently most important resistance mechanisms in *Enterbacteriaceae*, which reduces the efficacy even of modern expanded-spectrum cephalosporins (except cephamycins and carbapenems) and monobactams is based on plasmid-mediated production of enzymes that inactivate these compounds by hydrolyzing their beta-lactam ring. Such resistance is encoded by an increasing number of different point-mutational variants, called extended spectrum beta-lactamases (ESBL), of classical broad-spectrum beta-lactamases (BSBL): most are derivates of TEM and SHV beta-lactamase families, whereas other groups, such as CTX-M, OXA, PER and VEB beta-lactamases have been described more recently [1]. The phenotypical difference between BSBLs and ESBLs is that the latter efficiently hydrolyze 3^rd^- and 4^th^-generation cephalosporins, in addition to penicillins and 1^st^ and 2^nd^ generation cephalosporins as the BSBLs are capable of. ESBLs are inhibited by clavulanic acid, sulbactam and tazobactam [2], a feature that is used (i) as a criterion for classification of β-lactamases and (ii) for diagnostic ESBL detection purposes. As a matter of growing concern, resistance caused by ESBLs is often associated with resistance to other classes of antibiotics like fluoroquinolones, aminoglycosides and trimethoprim-sulfamethoxazole [3], [4].

Since the first description of ESBL producing *Enterobacteriaceae* isolated from hospitalized humans [5], many nosocomial outbreaks have been reported. However, since a few years, there is an increase in the detection of ESBL producing strains in the community [6], [7]. More recently, some reports have alerted about the dissemination of ESBL producing *E. coli* in healthy cattle in several countries in Europe and USA [8], [9], [10] or in cattle derived food products like meat and raw milk [11], [12], [13]. Therefore, the impact of healthy farm animals as a reservoir for an input of ESBL producing *E. coli* in the food processing chain has to be assessed.

The aim of the present study was to assess the prevalence of ESBL producing *Enterobacteriaceae* in the Swiss cattle population younger than two years, and to assess risk factors for shedding ESBL producing *Enterobacteriaceae* based on a slaughterhouse monitoring approach that is aiming at achieving a geographically representative sample. Whilst for monitoring purposes a geographically representative set is easily achieved for samples taken on a farm level, where farms to be sampled can be determined in advance by applying a stratified randomization scheme, this is typically more difficult for samples taken at the slaughterhouse.

## Materials and Methods

### Sampling

Representative samples for the cattle population younger than 2 years were taken at the slaughterhouse level. A minimum required sample size of 385 randomly selected animals was calculated with the assumptions of an infinite population size, a prevalence of 50%, a desired confidence level of 95.0% and an absolute error of 5% (Win Episcope 2.0 software http://www.clive.ed.ac.uk/winepiscope). The samples were randomly taken at the five biggest cattle abattoirs (A: Zurich; B: Hinwil; C: St. Gallen; D: Oensingen; E: Estavayer-le-Lac), where over 75% of Swiss cattle of the targeted age group are slaughtered. Because animals originating from farms located south of the Alps are typically underrepresented in the slaughter population of these 5 biggest abattoirs, samples from the biggest abattoir in the canton of Ticino (F: Bellinzona) and samples from several smaller abattoirs from the canton of Valais were additionally collected to guarantee a geographically representative distribution. From all the abattoirs we obtained the permission to use these animal parts.

Only one sample was taken per animal holding of origin. The number of samples in the sampling frame collected from each slaughterhouse was proportional to the number of cattle slaughtered at each establishment per year. Based on these data, a random sampling plan was conceived.

Faecal swabs were collected from November 2010 to September 2011 from 571 healthy animals younger than 2 years at slaughter. These animals had no contact to older cattle populations on the transport to the slaughterhouse, in the slaughterhouse or during the slaughtering procedure. Therefore, contamination by animals of older age classes after having left the farm of origin can be excluded. The samples were taken through opening the large intestine with sterile scissors after evisceration. Unique animal identification numbers registered in the Swiss central animal movement database (AMD) were recorded.

### Microbiological analysis

Each swab was incubated for 24 hours at 37°C in 10 ml EE Broth (BD, Franklin Lakes, USA) for enrichment. One loop of the enriched faecal samples were inoculated onto Brilliance ESBL agar (Oxoid, Hampshire, UK) and incubated at 37°C for 24 hours under aerobic conditions. All grown colonies were selected and sub-cultured onto Triple Sugar Iron (TSI) agar (BD, Franklin Lakes, USA) at 37°C for 24 hours. By the oxidase test and the assessment of lactose fermentation, non-fermenters were discarded, and oxidase-negative colonies were subjected to identification by API ID 32 E (bioMérieux, Marcy l'Etoile, France).

### Antimicrobial susceptibility testing and ESBL detection

All isolated strains were subjected to susceptibility testing for 18 antimicrobial agents by the disc diffusion method and evaluated according to CLSI criteria [14]. Strains exhibiting intermediate resistance were classified as susceptible. The antibiotics tested were: ampicillin (AM, 10 μg), amoxicillin/clavulanic acid (AMC, 20/10 μg), cephalothin (CF, 30 μg), cefuroxime (CXM, 30 μg), cefoxitin (FOX, 30 μg), cefpodoxime (CPD, 10 μg), cefotaxime (CTX, 30 μg), ceftazidime (CAZ, 30 μg), cefepime (FEP, 30 μg), ciprofloxacin (CIP, 5 μg), nalidixic acid (NA, 30 μg), gentamicin (GM, 10 μg), streptomycin (S, 10 μg), trimethoprim-sulfamethoxazole (SXT, 1.25/23.75 μg), chloramphenicol (C, 30 μg) and tetracycline (TE, 30 μg). The amoxicillin/clavulanic acid disc was placed between the cefpodoxime and the ceftazidime discs, and the synergy effect was documented. The strains, which showed a synergy effect, were then confirmed as ESBL producers on Muller-Hinton agar plates using E-Test-ESBL strips containing cefotaxime, cefepime or ceftazidime alone and in combination with clavulanic acid (bioMérieux, Marcy l'Etoile, France).

### Risk Factor Analysis

Risk factors were calculated based on data obtained from the Swiss AMD. The individual animal identification number of the slaughtered and sampled animals served as a unique identifier to determine the farm of origin, date of birth and to access all incoming movement data corresponding to the farm of origin and the length of stay of each individual animal of the sample. Risk factors derived from AMD data comprised age, main production type on the farm of origin (dairy vs. beef and fattening), farm size, number of animal movements and number of animals found death or euthanized on farm. The full list of studied risk factors and their categorization is given in Table 1.

**Table 1:** Description of risk factors studied in univariate and multivariate logistic regression models.

Risk factor	Category	Number of animals	Proportion of positive samples
Age Class	≤180 d	248	15.7%
	181 d–1 y	148	3.4%
	>1 y	175	2.3%
Production Type	Meat	244	2.5%
	Dairy	327	12.8%
Number of cattle on farm	≤30	173	6.4%
	31–60	211	8.1%
	>60	187	10.7%
Animal movements to farm	≤0.5	428	6.3%
per day	>0.5	143	14.7%
Animal movements to farm	≤1/d/100 animals	461	6.9%
per day per 100 animals	>1/d/100 animals	110	14.6%
Number of animals dying	0	235	10.2%
on farm per 100 days	≤1/100 d	185	4.3%
	>1/100 d	151	10.6%
Number of animals dying on	0	235	10.2%
farm per 100 days per 100	≤2/100 d	200	4.0%
animals	>2/100 d	136	11.8%

### Statistical Analysis

Statistical analyses were performed in R version 2.13 for Mac OS. The significance level was set at p≤0.05. ESBL prevalence and their Yates' continuity corrected 95% confidence intervals were calculated applying exact binomial tests. Logistic regression models were applied for risk factor analyses. First, a univariate model was calculated for each risk factor given in Table 1. Risk factors with a p-value <0.25 were retained for multivariate analyses. To avoid colinearity, the correlation structure of these retained risk factors was assessed. Of each pair of correlating risk factors, only the one showing the more significant association with the dependent variable was retained for the multivariate model. Multivariate models were fitted by backward elimination procedures. According to Hosmer and Lemeshow [15] confounders were eliminated if they did not importantly change the estimates of the significant predictors. All two ways interactions were tested.

## Results

The sampling regimen resulted in a geographically representative sample population that was uniformly spread over the entire territory of Switzerland. Farms of origin of cattle shedding ESBL-producing *Enterobacteriaceae* are as well uniformly spread over the densely populated Swiss midlands as well as over the canton of Ticino in the south east of Switzerland. There were no cattle shedding ESBL-producing *Enterobacteriaceae* found originating from the canton of Valais in the south west of Switzerland.

ESBL-producing *Enterobacteriaceae* were detected in 48 (8.4%; 95% C.I. 6.3–11.1%) of the 571 tested animals. ESBL prevalence in different age classes, different production types and different animal movement activities in the farm of origin are summarized in Table 2. Results of the final multivariate logistic regression models applied for risk factor analysis are shown in Tables 3 and 4. Animals from within the age class ≤180 days were at a significantly higher risk of shedding ESBL-producing *Enterobacteriaceae* than animals from age classes 180 days to 1 year or 1 year to 2 years. Animals originating from farms with primary production type „dairy“ were at a 5.95 times greater risk of shedding ESBL-producing *Enterobacteriaceae* than animals originating from farms with primary production type „beef“. Finally, animals originating from farms with more than one animal movement per day per 100 animals were at a 2.37 times higher risk of shedding ESBL-producing *Enterobacteriaceae* than animals originating form farms with less than one animal movement per day100 animals.

**Table 2:** Prevalences of animals with ESBL positive isolates.

	No. positives	No. negatives	prevalence	95% confidence interval *
Total	48	523	8.41%	6.32–11.07%
≤180 d	39	209	15.73%	11.55–21.00%
181 d–1 y	5	143	3.38%	1.25–8.12%
>1 y	4	171	2.29%	0.73–6.12%
Meat	6	238	2.46%	1.00–5.53%
Dairy	42	285	12.84%	9.51–17.08%
≤1 mv./d/100 animals	32	429	6.94%	4.87–9.76%
>1 mv./d/100 animals	16	94	14.55%	8.80–22.85%

* 95% confidence interval with Yates' continuity correction.

**Table 3:** Farm level risk factors for ESBL shedding.

Risk factor		OR	95% Conf. Int.		p-value
Prod. type	meat	1.00			
	dairy	5.95	2.48 -	14.30	<0.001
Movements	≤1/d/100 anim.	1.00			
	>1/d/100 anim.	2.37	1.23 -	4.57	0.010

Multivariate logistic regression model.

**Table 4:** Animal level risk factors for ESBL shedding.

Risk factor		OR	95% Conf. Int.		p-value
Age	≤180 d	1.00			
	181 d–1 y	0.19	0.07 -	0.49	<0.001
	>1 y	0.12	0.04 -	0.36	<0.001

Logistic regression model.

**Figure 1:**
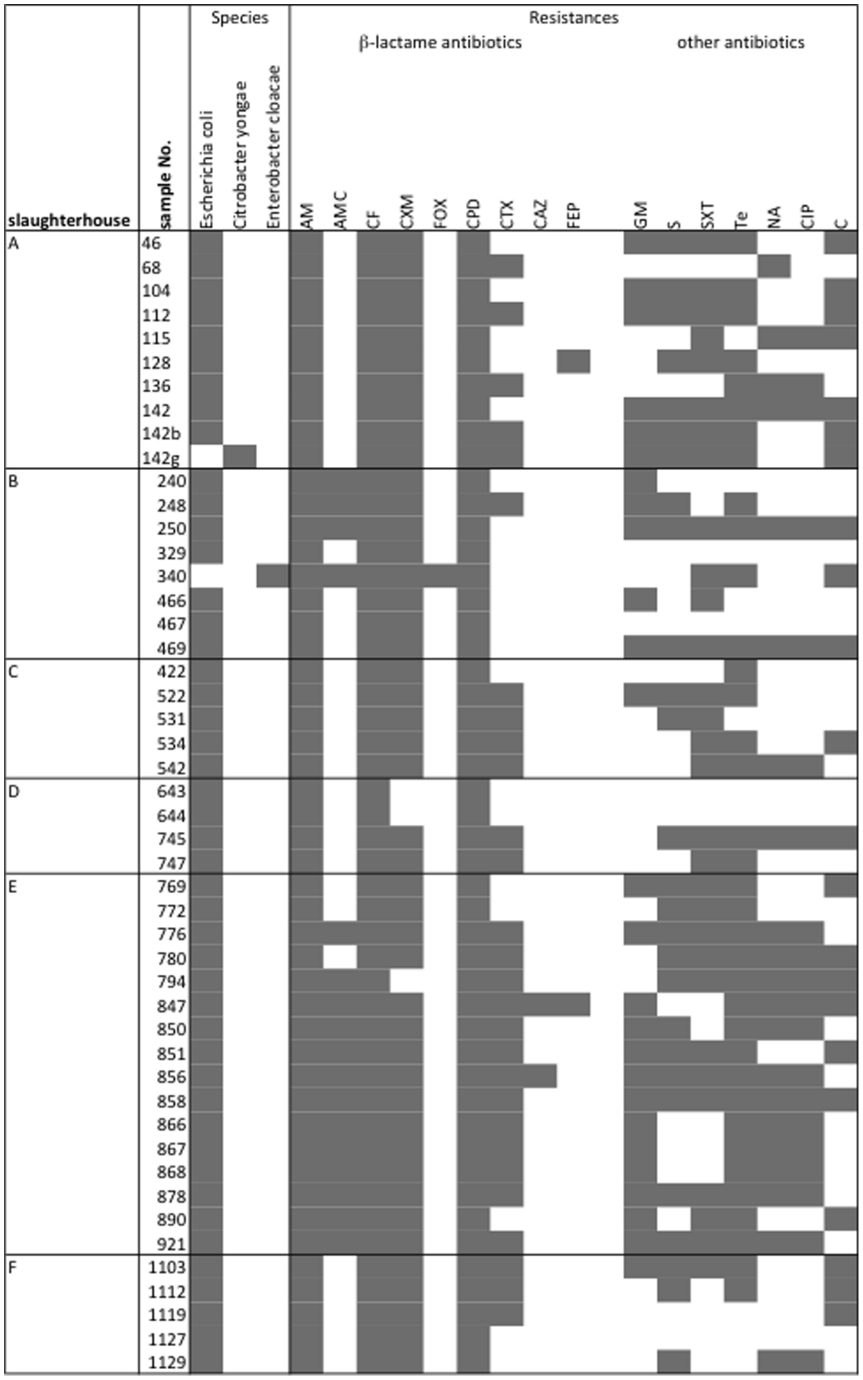
Characteristics and antimicrobial susceptibility profiles of ESBL-producing *Enterobacteriaceae* isolated from cattle younger than 2 **years.** Symbols: black square, positive result or resistant to a specific antimicrobial agent; white square, negative result or susceptible to a specific antimicrobial. Abbreviations: AM, ampicillin (resistant ≤13 mm); AMC, amoxicillin-clavulanic acid (resistant ≤13 mm); CF, cephalothin (resistant ≤14 mm); CXM, cefuroxime (resistant ≤14 mm); FOX, cefoxitin (resistant ≤14 mm); CPD, cefpodoxime (resistant ≤17 mm); CTX, cefotaxime (resistant ≤14 mm); CAZ, ceftazidime (resistant ≤14 mm); FEP, cefepime (resistant ≤14 mm); GM, gentamicin (resistant ≤10 mm); S, streptomycin (resistant ≤11 mm); SXT, trimethoprim-sulfamethoxazole (resistant ≤10 mm); TE, tetracycline (resistant ≤11 mm); NA, nalidixic acid (resistant ≤13 mm); CIP, ciprofloxacin (resistant ≤15 mm); C, chloramphenicol (resistant ≤12 mm). Discrimination between “susceptible” and “resistant” was strictly according to CLSI interpretive criteria. It should be noted, however, that for clinical and therapeutic purposes, ESBL producers should generally be reported resistant to cephalosporins of all 4 generations and monobactams.

In total, 48 isolates from 48 different animals showing the ESBL phenotype were further characterized. Species identification revealed 46 *E. coli*, one *Enterobacter cloacae* and one *Citrobacter youngae* (Figure 1). In view of β-lactam antibiotics, all 48 isolates were resistant to ampicillin (AM), the 1^st^-generation cephalosporin, cephalothin (CF) and the 3^rd^-generation cephalosporin, cefpodoxime (CPD) (Figure 1). Forty-five (93.8%) isolates were resistant to the 2^nd^-generation cephalosporin cefuroxime (CXM); one (2.1%) isolate to cefoxitin (FOX), 28 (58.3%) isolates to cefotaxime (CTX), 2 (4.2%) isolates to ceftazidime (CAZ), and 2 (4.2%) isolates to the 4^th^-generation cephalosporin cefepime (FEP). Seventeen strains (35.4%) showed resistance to amoxicillin-clavulanic acid (AMC). Besides β-lactam resistance, susceptibility to other classes of antibiotics was tested. Hereby, 26 (54.2%) isolates were resistant to gentamicin (GM), 27 (56.25%) to streptomycin (S), 21 (43.8%) to nalidixic acid (NA), 20 (41.7%) to ciprofloxacin (CIP), 36 (75.0%) to tetracycline (TE), 22 (45.8%) to chloramphenicol (C), and 30 (62.5%) to trimethoprim-sulfamethoxazole (SXT).

## Discussion

In this cross-sectional study, fecal samples of a geographically representative cattle subpopulation for Switzerland were sampled at slaughterhouse level to determine the occurrence of ESBL producing *Enterobacteriaceae* in the Swiss cattle population younger than 2 years, and to assess risk factors for shedding such organisms.

To assess the population prevalence of a pathogen or indicator organism on a national level, a geographically representative set of samples needs to be selected. Whilst this is easily achieved for samples taken on a farm level, where farms to be sampled can be determined in advance by applying a stratified randomization scheme, this is typically more difficult for samples taken at the slaughterhouse. This study demonstrated that the goal of a geographically representative survey could be achieved with a carefully planned sampling scheme involving the major cattle slaughterhouses and additional small abattoirs in areas disconnected from the main animal traffic.

To our knowledge, this is the first study on risk factors for shedding ESBL producing *Enterobacteriaceae* working with risk factors derived from animal movement data stored in a national AMD. Performing risk factor analysis with such data offered several advantages compared to risk factors derived form surveys, especially when the samples were collected at the abattoir and not on the farm. Getting information on animal movements and mortalities from 571 farms of origin of slaughtered animals via telephone or email surveys would be very time consuming. Furthermore, such information would be very imprecise compared to data from the Swiss AMD whose content is very accurate and complete.

The overall prevalence of cattle hosting ESBL producing *Enterobacteriaceae* found in this study (8.4%; 95% C.I. 6.3–11.1%) is slightly lower than found in previous, smaller scaled Swiss studies that reported prevalences of 13.7% [16] and 17.1% [10]. Recent studies from other countries reported a very low (0.2%) prevalence in cattle in Korea [17] or even zero prevalence in cattle in Tunisia [18]. Studies from European countries reported 35.4% (22.2–50.5% C.I. 95%) in North West England and North Wales [19] and 4.8% in France [20].

One of the significant risk factors for shedding ESBL producing *Enterobacteriaceae* obtained in the final multivariate model was age, with cattle over 6 months being at a considerably lower risk than calves (younger than 6 months) This is in accordance with a previous study of Geser et al. [16] who reported a overall prevalence of 13.7% in cattle as opposed to a prevalence of 25.3% among calves.

Animals from dairy farms were at a 5.95 times higher risk for hosting ESBL producing *Enterobacteriaceae* than animals from beef or fattening farms. This might be explained by differences between production types with respect to farming practices and antimicrobial compounds applied. In Switzerland, sales of cephalosporins have increased over the past years, especially the sales of 3^rd^ and 4^th^ generation cephalosporins for intramammary application [21]. Snow et al. [19] reported that in North West England and North Wales farms that had used 3^rd^ or 4^th^ generation cephalosporins in livestock during the previous 12 months were nearly 4 times more likely to host ESBL *E. coli*. On Swiss dairy farms, calves not in consideration for breeding are either fattened on their farm of birth or they are sold to fattening farms at a very young age. Those fattened on the dairy farms are primarily fed with milk. For economic reasons, milk that cannot be put on the market because of elevated cell counts or because of recent antimicrobial treatments is often fed to calves. Moreover, ESBL hosting *Enterobacteriaceae* present on dairy farms might be transmitted to calves by the fecal-oral route. Calves later to be fattened on fattening farms leave their dairy farms very few weeks after birth and are thus at a lower risk for acquiring ESBL producing *Enterobacteriaceae* than animals staying on the dairy farms until slaughter. In comparison to dairy farms, beta-lactam antibiotics, especially 3^rd^ and 4^th^ generation cephalosporins, do not represent the predominantly used antimicrobial compounds on beef and fattening farms. The fact that among 40 animals originating from the canton of Valais no ESBL producers were found, might also be explained by the farming type, as meat production is predominant over milk production in this area.

The number of animal movements per farm per day per 100 animals is a factor related to introduction of new stock, and it was associated with presence of ESBL *Enterobacteriaceae*. Animals from farms with a high number of animal incoming movements in relation to total farm size were at a 2.37 times higher risk of hosting ESBL producing *Enterobacteriaceae* than animals from farms with a lower level of animal traffic. This is in agreement with Snow et al. [19] who reported that operating a closed farm policy reduced the risk of the farm having ESBL *E. coli* compared to farms that were open and did not quarantine new cattle.

The confirmation of relatively high rates of ESBL producers in cattle and the high diversity among the isolates are worrisome and indicate an established reservoir, especially in dairy farms. A prudent use of antibiotics, especially of 3^rd^ and 4^th^ generation cephalosporins, restrictions in feeding milk of treated cows to calves and management improvements to facilitate the operation of closed herd policies could represent modes of action towards reduction of ESBL prevalence in cattle. Experimental studies would be needed to assess the effectiveness of such measures. Moreover, this study showed that with a carefully planned sampling scheme involving the major cattle slaughterhouses and – in addition – small abattoirs in areas disconnected from the main animal traffic, geographically representative surveys can be achieved by taking random samples at the abattoir.
